# Insecticide resistance status predetermines behavioural responses and oviposition fitness in *Anopheles coluzzii*

**DOI:** 10.1371/journal.pone.0360986

**Published:** 2026-10-15

**Authors:** Ehimwenma Aghahowa, Victor N. Enwemiwe, Eric Esiwo, Precious O. Edhebe, Sarah E. Ifeta, Chioma C. Ojianwuna

**Affiliations:** Department of Animal and Environmental Biology, Faculty of Science, Delta State University, Abraka, Nigeria; World Health Organization, Regional Office for South-East Asia, INDIA

## Abstract

Behavioural resistance is one of the major public health challenges facing planned vector control strategies involving insecticides. Understanding the behaviour of mosquitoes holistically when exposed to insecticides could be key to adding up to the fight against resistance. This study evaluated the behavioural responses and oviposition fitness of male and female *Anopheles coluzzii* exposed to selected pyrethroids, including lambda-cyhalothrin (0.05%), permethrin (0.75%), and DDT (4%). Male and female mosquitoes, aged three to five days, were exposed in eight replicates to selected insecticides for 60 minutes. Susceptibility to insecticide exposure was assessed in a total of 2,000 mosquitoes (eight replicates of 25 per sex per insecticide, across three insecticides), while behavioural assays used resistant and unexposed cohorts of 15 mosquitoes per replicate, in triplicate, for three insecticides (lambda-cyhalothrin, permethrin, and DDT). Resistant male and female mosquitoes were separated in holding buckets, tagged, sugar-fed, and kept to acclimatize for 1 hour before further exposure for the behavioural study. Square-shaped containers connected by a pipe into an H-shape were used for the experiment. Resistant mosquitoes (n = 15) were exposed to different chambers with insecticide-treated paper (WITP) and without insecticide-treated paper (WOITP) in triplicate. A similar experiment was done for unexposed mosquitoes. The flight efficiency, resting efficiency, repellency, landing, and biting efficiency of mosquitoes were recorded. Sex crossing between exposed and unexposed mosquitoes was done, and oviposition fitness was assessed. Flight efficiency of resistant mosquitoes WITP increased (mean 3.00–30.5) whereas WOITP decreased (mean 2.00–0.50) significantly (p < 0.0001). Resting efficacy WITP decreased (mean 12.50–0.00) while WOITP increased (mean 11.50–13.50) significantly (p < 0.0001). Repellency WITP decreased (mean 14.00–0.00), while WOITP did not follow a regular pattern. Likewise, landing and biting did not follow a regular pattern. Unexposed female mosquitoes had the highest blood meal (mean of 11.5). The highest ovipositioning was recorded in unexposed males and females (mean of 0.33), but no ovipositioning was recorded in other crosses (mean of 0.00). Insecticide exposure induces both behavioural avoidance and reproductive impairment in resistant *Anopheles coluzzii*, emphasizing the importance of incorporating behavioural endpoints into resistance monitoring programs.

## Introduction

Malaria remains one of the most persistent and devastating vector-borne diseases globally, with a disproportionate burden in tropical and subtropical regions. Sub-Saharan Africa continues to account for the majority of malaria morbidity and mortality, with Nigeria contributing nearly one-quarter of global cases [1]. Despite notable progress achieved through coordinated vector control interventions over the past two decades, malaria transmission remains sustained in many endemic settings, highlighting the limitations of current control strategies [World Health Organization, 2025]. Female mosquitoes of the genus *Anopheles* mediate transmission, and *Anopheles coluzzii* has emerged as a highly efficient and adaptable vector across West Africa [2,3]. Its strong anthropophilic and endophilic tendencies have historically made it a key target of indoor-based control interventions.

Vector control remains the cornerstone of malaria prevention and relies predominantly on insecticide-based tools such as long-lasting insecticidal nets (LLINs) and indoor residual spraying (IRS) [4,5]. These interventions have been highly effective due to their ability to reduce human–vector contact and induce mosquito mortality. Pyrethroids, in particular, have been widely adopted because of their rapid knockdown effects, excito-repellent properties, and relatively low toxicity to humans [6]. However, the intensive and prolonged application of these insecticides has imposed strong selective pressures on mosquito populations, resulting in the widespread emergence of resistance across Africa [6,7]. This resistance threatens to reverse gains achieved in malaria control and presents a major challenge to elimination efforts.

Insecticide resistance in malaria vectors is a multifactorial phenomenon involving a combination of physiological, biochemical, and genetic mechanisms. These include enhanced metabolic detoxification mediated by enzymes such as cytochrome P450 monooxygenases, glutathione S-transferases, and esterases; target-site insensitivity resulting from mutations in key genes such as the voltage-gated sodium channel (kdr mutations); and reduced insecticide penetration due to cuticular modifications [6,8]. Among these, kdr mutations are particularly well documented and are strongly associated with resistance to pyrethroids and DDT, reducing neuronal sensitivity and allowing mosquitoes to survive exposures that would otherwise be lethal [9]. While these mechanisms have been extensively studied, they do not fully explain the persistence of malaria transmission in areas with high coverage of vector control interventions.

Increasing evidence suggests that behavioural resistance plays a complementary and potentially equally important role in the survival of mosquito populations under insecticide pressure. Behavioural resistance refers to adaptive modifications that reduce contact with insecticides, including avoidance of treated surfaces, changes in resting and feeding behaviour, shifts in biting time, and increased flight activity [5,10,11]. Unlike physiological resistance, which allows mosquitoes to survive after contact, behavioural resistance enables them to avoid exposure altogether. These changes can significantly compromise the effectiveness of LLINs and IRS, as mosquitoes may increasingly feed outdoors, bite earlier in the evening, or avoid treated resting surfaces [5]. For consistency, the term “behavioural resistance” is used throughout this manuscript to refer to these non-genetic behavioural mechanisms that reduce mosquito-insecticide contact. Such behavioural plasticity enables continued malaria transmission even in settings with high intervention coverage.

The adaptive capacity of *Anopheles coluzzii* further complicates control efforts. This species exhibits remarkable ecological flexibility, allowing it to persist across diverse environmental conditions in West Africa [3,12]. Although traditionally characterized as endophilic and anthropophilic, recent studies indicate shifts in behaviour that may reduce its susceptibility to indoor interventions [13–15]. In Nigeria, high levels of resistance to pyrethroids and DDT have been documented, driven by both metabolic and target-site mechanisms [8,13,16]. However, most studies have focused primarily on mortality outcomes from standard susceptibility assays, with limited attention given to behavioural endpoints such as flight dynamics, resting behaviour, repellency, and host interaction. This represents a critical gap in understanding how resistant populations persist and continue to transmit malaria under sustained insecticide pressure.

Beyond behavioural resistance, insecticide resistance may also influence mosquito fitness, with important implications for population dynamics and the spread of resistance traits. Fitness costs associated with resistance can include reduced fecundity, impaired mating success, altered feeding efficiency, and decreased survival [17–19]. However, findings remain inconsistent, with some studies reporting minimal or no reproductive penalties, suggesting that certain resistance mechanisms may confer adaptive advantages without significant trade-offs [6,7]. Notably, most investigations have focused on female mosquitoes, given their direct role in disease transmission, while male behavioural responses and their contribution to resistance propagation remain largely understudied. Emerging evidence of phenotypic resistance in male populations [20] highlights the need to incorporate both sexes into behavioural and reproductive studies.

Furthermore, the interaction between behavioural responses and reproductive fitness remains poorly understood. Sublethal exposure to insecticides may alter not only immediate behavioural traits such as flight and resting but also longer-term biological processes including mating success and oviposition. These combined effects may influence both vectorial capacity and the evolutionary trajectory of resistance within mosquito populations. Understanding these interactions is particularly important in endemic regions such as Delta State, Nigeria, where environmental conditions favour continuous mosquito breeding and repeated exposure to insecticides.

The WHO Global Plan for Insecticide Resistance Management (GPIRM) provides a coordinated strategic framework for addressing the growing threat of insecticide resistance in malaria vectors, emphasizing the need for integrated resistance monitoring that extends beyond physiological susceptibility testing [21]. Complementing this, conceptual frameworks for behavioural resistance surveillance highlight the importance of pairing physiological susceptibility monitoring with assessments of behavioural phenotypes that determine the real-world impact of vector control interventions [22]. Recent field studies have further demonstrated the emergence of behavioural avoidance strategies, such as shifts in resting location and host choice, in malaria vector populations under sustained insecticide-based control pressure [23].

Given these knowledge gaps, there is a need for integrated studies that simultaneously assess behavioural responses and reproductive fitness in resistant mosquito populations. Therefore, this study evaluated the influence of insecticide resistance on key behavioural parameters, including flight activity, resting behaviour, repellency, landing, and biting efficiency, as well as oviposition fitness in both male and female *Anopheles coluzzii* exposed to lambda-cyhalothrin, permethrin, and DDT. By combining behavioural and reproductive endpoints, this study aims to provide a more comprehensive understanding of how resistance shapes mosquito adaptation and to inform the development of more effective and sustainable vector control strategies.

## Materials and methods

### Study design

This study employed a controlled experimental design to investigate how prior insecticide exposure influences behavioural responses and reproductive fitness in Anopheles coluzzii. The study integrates phenotypic resistance selection with behavioural and reproductive assays to capture both immediate and downstream effects of insecticide exposure. Specifically, mosquitoes that survived standard insecticide exposure after 24 hours (hereafter referred to as “resistant species surviving insecticide exposure”) were compared with unexposed cohorts to evaluate differences in flight activity, resting behaviour, repellency, landing and biting efficiency, and oviposition success. This design allows for the assessment of both behavioural resistance traits and fitness consequences, which are often overlooked in conventional susceptibility assays.

### Study area

The study was conducted in Delta State, located in the Niger Delta region of southern Nigeria. Mosquito larvae were collected from multiple Local Government Areas, including Ethiope East (5.61°N, 5.81°E), Ukwuani (5.70°N, 6.20°E), Isoko North (5.50°N, 6.25°E), and Okpe (5.90°N, 5.68°E). The region lies within the tropical rainforest belt and is characterized by high humidity, elevated temperatures, and distinct wet and dry seasons. Abundant aquatic habitats such as swamps, rivers, and stagnant pools provide ideal breeding conditions for *Anopheles* species, supporting perennial malaria transmission. The primary economic activities of residents in these communities are farming and petty trading.

### Mosquito collection and rearing

Immature stages (larvae and pupae) of malaria vectors were collected from identified breeding sites using standard dipping techniques in accordance with [24]. Collections were carried out during the rainy season, from April to July 2025, on randomly selected days within this period. Collected specimens were transported to the Entomology Unit insectary at Delta State University, Abraka, where they were reared under controlled laboratory conditions. Larvae were maintained in plastic trays and fed a standardized diet consisting of finely ground low-fat biscuits supplemented with yeast. Upon emergence, adult mosquitoes were transferred into holding cages and maintained on 20% honey solution. Larvae and adults were reared under controlled insectary conditions at 28 ± 2°C and 85% relative humidity. Adults aged between three and five days post-emergence were used for all experimental procedures to ensure physiological uniformity.

### Molecular identification of mosquitoes

Mosquito DNA was extracted and amplified using the PCR method. The protocol for extraction of genomic DNA by [25] was strictly followed. The boiling method was used for ribosomal DNA extraction. Mosquitoes were housed in the microcentrifuge tubes (UN1–50), which were labeled. The Lysis Buffer (500µl) was introduced into the tube. Mosquitoes were crushed using the Kento pestle. The contents of each tube were mixed using a vortex mixer and incubated at 56^0^C for 10 minutes. More so, centrifugation was done at 10,000 rpm for a few seconds to separate contents. Absolute ethanol (200µl) was added to each tube after spinning. The mix was transferred to a spin column, mixed thoroughly by inversion, and further centrifuged at 10,000 rpm for 30 seconds. The process was repeated at different times to obtain the pure DNA of the mosquitoes, which was stored at −2^00^C for amplification by PCR. Primer designs were extracted from sequenced ribosomal RNA gene of *An. gambiae sl* [26].

PCR method by [26] was used for the identification of subspecies of *An. gambiae* s.l. The PCR- restriction fragment length polymorphism (RFLP) diagnostic test [27] and SINE 200 PCR method by [28] were used to identify genomic DNA from *Anopheles gambiae s.s.* and *An*. *coluzzii*. Specific primers included for *Anopheles gambiae* (5-GCTTACTGGTTTGGT CGGCATTG-3), *Anopheles arabiensis* (5-GTGTTAAGTGTCCTTCTCCGTC-3), and *An. quadriannulatus* (5-GCATGTCCAAGATGGTTCGCTG-3), *Anopheles colluzzi* (M form; 5-TAGCCAGCTCTTGTCCACTAGTTTT-3), *Anopheles sensu strecto* (S form; 5-CCAGACCAAGATGGTTCGCTG-3). PCR was done with initial denaturation at 95℃ for 5 min (1 cycle), denaturation at 95℃ for 30 sec, annealing at 59.2℃ for 30 sec followed by extension at 72℃ for 30 sec (30 cycles), and final extension at 72℃ for 5 min (1 cycle) with final hold at 4℃.

### Insecticide exposure and selection

Adult male and female mosquitoes were separated and subjected to insecticide exposure assays using [24]. The insecticides tested included lambda-cyhalothrin (0.05%), permethrin (0.75%), and DDT (4.00%). These insecticides were selected to represent the organochlorine class (DDT) and the two most commonly used pyrethroids in local vector control programmes (permethrin and lambda-cyhalothrin). Mosquitoes were exposed for 60 minutes in eight replicates per insecticide treatment. Knockdown was not recorded, as the focus of the study was on post-exposure behavioural outcomes rather than acute toxicity. After 60 minutes of exposure to insecticide, surviving mosquitoes were held for 24 hours and classified using WHO standard method of insecticide susceptibility as confirmed species resistance after insecticide exposure and transferred into labelled holding containers according to sex and treatment group. Mosquitoes surviving insecticide exposures were provided with sugar meals and allowed a 1-hour acclimatization period prior to behavioural assays. This study adopted the WHO bioassay method and selected mosquitoes following phenotypic resistance to a single diagnostic dose of insecticides. Mosquitoes surviving the insecticides were those alive after 24 hours of post-exposure holding and alive after acclimatization for 1hr. Dead mosquitoes were discarded as they were irrelevant to this study’s objectives. Unexposed mosquitoes maintained under identical conditions served as controls.

Further to determining the baseline phenotypic resistance of the mosquito population, the mosquitoes exposed to DDT (organochlorine, 4.00%) and the pyrethroids permethrin (0.75%) and lambda-cyhalothrin (0.05%) using the WHO standard susceptibility bioassays were classified. Populations were classified as susceptible (≥98% mortality), possibly resistant (90–97% mortality, requiring confirmation), or resistant (<90% mortality), following [24] criteria.

### Behavioural assay

The experimental apparatus consisted of two square transparent plastic chambers (Zone A and Zone B) positioned side by side and connected via a short horizontal conduit, forming an H-shaped configuration, with dimensions matching the protocol described by [29] (Fig 1). Each chamber had a mesh-covered lid permitting visual observation of mosquito behaviour from the top and sides, and a separate circular mesh-covered port on the side of the chamber through which mosquitoes were introduced by hand or aspirator. At the start of each trial, mosquitoes were collected from the holding cage using an aspirator and released into Zone A, which contained the insecticide-treated paper; Zone B remained untreated, allowing assessment of escape and repellency behaviour as mosquitoes moved from the treated zone into the untreated zone through the connecting conduit. Trials were illuminated using a ring light positioned adjacent to the apparatus to enable clear observation and recording of mosquito activity. Control trials used an identical A–B setup run simultaneously but with no insecticide present in either zone, providing a baseline for behavioural variables in the absence of an insecticidal stimulus. Two experimental conditions were established: with insecticide-treated paper (WITP) and without insecticide-treated paper (WOITP). For each treatment, groups of 15 mosquitoes were introduced into the system, with experiments conducted in triplicate for both resistant and unexposed populations. Behavioural parameters, including flight efficiency, resting efficiency, repellency (movement away from treated zones), landing frequency, and biting activity, were recorded over defined time intervals from 10 to 60 minutes. This setup enabled the quantification of behavioural avoidance, excitability, and host interaction dynamics under insecticide exposure gradients. Flight, resting, and repellency assays were conducted using only female mosquitoes, given their primary role as the sole blood-feeding sex and vectors of malaria parasites. Landing and biting assays included both male and female mosquitoes to determine whether males exhibited any blood-feeding-associated behaviour despite their non-vector status.

**Fig 1:**
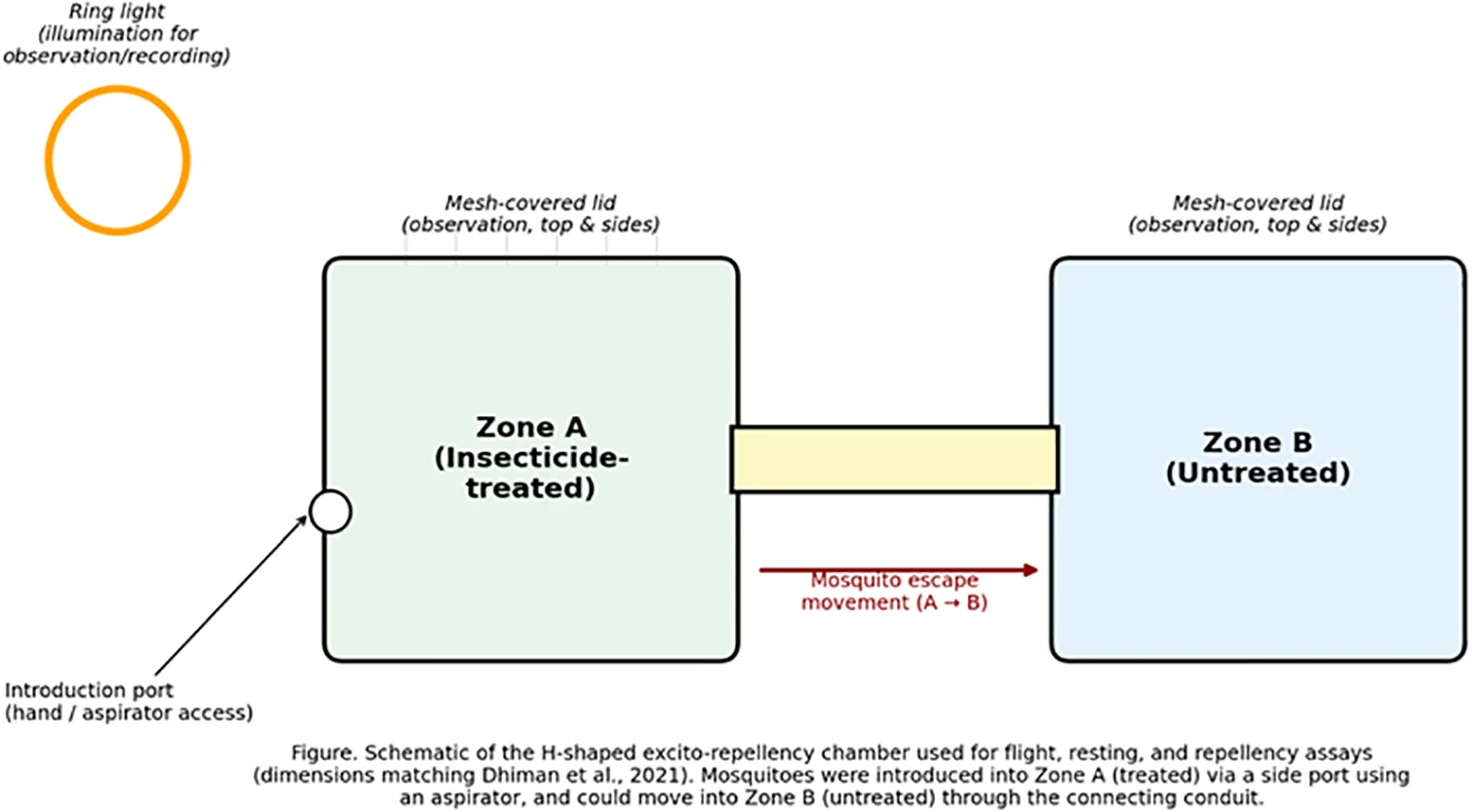
Schematic of the H-shaped excito-repellency chamber. Zone A = insecticide-treated; Zone B = untreated, showing the connecting conduit, mesh-covered observation lids, side introduction port, and ring-light illumination used during flight, resting, and repellency assays. Dimensions matched the protocol of [29].

### Definitions of behavioural outcome measures

To ensure accurate tracking of each behavioural outcome, a separate trial (with a new exposure and a new video recording) was conducted for each parameter measured (flight, resting, repellency, landing, biting), with the observer(s) focused solely on that outcome during video review. This approach was adopted to avoid loss of tracking accuracy when monitoring multiple behavioural variables simultaneously. All trials were video-recorded and reviewed by the lead observer together with four assistant observers.

Flight efficiency was defined as the cumulative number of flight (take-off) events recorded per group within each 10-minute interval, expressed as a percentage using the formula.


$$\begin{array}{c} \text{Flight efficiency }(\%)\;=\frac{Number\;of\;mosquitoes\;flights}{Total\;number\;of\;mosquitoes\;flights\;}x\text{1}\text{0}\text{0} \end{array}$$

Resting efficiency was defined as the cumulative number of resting events recorded per group within each 10-minute interval. A resting event was recorded when a mosquito settled and remained stationary (perched) on an interior surface of the chamber, expressed as a percentage using the formula.


$$\begin{array}{c} \text{Resting efficiency }(\%)\;=\frac{Number\;of\;mosquitoes\;rests}{Total\;number\;of\;mosquitoes\;rests}x\text{1}\text{0}\text{0} \end{array}$$

Flight and resting were recorded as the only two behavioural states during these assays; no alternative state (e.g., walking or stationary-but-not-resting) was recorded. Accordingly, flight and resting were mutually exclusive and complementary within each observation interval, such that an increase in one is expected to correspond to a decrease in the other.

Repellency was defined as the cumulative number of escape events recorded per group within each 10-minute interval, where an escape event was recorded when a mosquito moved from Zone A (treated) into Zone B (untreated) through the connecting conduit, expressed as a percentage using the formula.


$$\begin{array}{c} \text{Repellency }(\%)\;=\frac{number\;of\;mosquitoes\;in\;control\;group\;-number\;of\;mosquitoes\;in\;treated\;group}{number\;of\;mosquitoes\;in\;control\;group}x\text{1}\text{0}\text{0} \end{array}$$

Landing was defined as the cumulative number of landing events recorded per group within each 10-minute interval, where a landing event was recorded when a mosquito perched (stood still) on the insecticide-treated paper surface, expressed as a percentage using the formula.


$$\begin{array}{c} \text{Landing }(\%)\;=\frac{Number\;of\;mosquitoes\;landing}{Total\;number\;of\;mosquitoes\;}x\text{1}\text{0}\text{0} \end{array}$$

Biting was defined as the cumulative number of biting events recorded per group within each 10-minute interval, where a biting event was scored when a mosquito’s proboscis penetrated the skin of a human hand (bait) to obtain a blood meal. The same human hand was introduced into the chamber immediately at the start of each biting trial and used consistently across all biting trials.

The number of resistant females available for landing and biting assays was fifteen (15) per replicate for the three insecticides selected. An equal number of resistant males were blown into the respective holding cages to match the corresponding female sample sizes.

### Oviposition fitness assessment

To evaluate the reproductive fitness of insecticide exposure, controlled mating experiments were conducted using combinations of exposed and unexposed male and female mosquitoes. Several crosses were carried out to assess the oviposition fitness of the mosquitoes. These included a cross between unexposed males and unexposed females (control), exposed females and unexposed males, and exposed males and unexposed females (for each insecticide type). The cross was done using five (5) mosquitoes (5 females and 5 males) designated from landing and biting exposures. The mosquitoes were maintained in a holding cage supplied with sugar solution for 48 hours to allow mating to occur, following the typical reproductive sequence in *Anopheles* in which virgin females take a sugar meal upon emergence before seeking a mate, with mating generally occurring within this period. Mating was inferred from subsequent blood-feeding and oviposition, consistent with the established sugar-feeding → mating → blood-feeding sequence in mosquito reproductive physiology; direct confirmation of insemination (e.g., via spermathecal dissection) was not performed, and this is acknowledged as a limitation of the study. The feeding proportion of the female mosquitoes was classified with reference to the abdomen by [30] as unfed (deflated abdomen with no blood meal), half fed (inflated anterior abdomen), and blood fed (inflated abdomen with blood meal). After mating and blood-feeding characterization under a glass aspirator, females were individually transferred into 2 mL oviposition chambers (Eppendorf tubes) lined with moist cotton wool and filter paper to facilitate egg laying. Blood-feeding success was assessed by visual inspection for abdominal engorgement immediately following the feeding opportunity under a glass aspirator, with each female checked once. Blood-feeding rate was calculated as the number of engorged females divided by the number of females offered a blood meal, expressed as a percentage, following standard entomological indices. A small perforation was introduced into each tube to ensure ventilation. Females were introduced using a manual aspirator and monitored for oviposition over the experimental period. Outcomes were measured according to the number of egg clusters laid and viability of eggs (where applicable).

### Field permit

Field site access has earlier been granted by the Delta State Ministry of Health and Delta State Ministry of Environment for the commencement of the Delta Sentinel site project. This was captured in the ethical permit document, which aided the approval of this study.

### Data analysis

Behavioural and oviposition fitness data were expressed as mean ± standard deviation. Differences between treatment groups and time intervals were analyzed using appropriate statistical tests, including analysis of variance (ANOVA), with significance set at *p* < 0.05. For flight, resting, and repellency, one-way ANOVA was used to compare mean values among the twelve exposure-condition combinations, defined by the three insecticides (DDT, permethrin, lambda-cyhalothrin), resistance status (resistant vs. unexposed), and exposure form (WITP vs. WOITP), collapsed across the observation period. For landing and biting, one-way ANOVA compared mean values among four treatment groups (control, lambda-cyhalothrin, permethrin, DDT). Where specifically noted, a separate one-way ANOVA was conducted on the percentage-transformed values shown in the corresponding figures.

## Results

### Insecticide bioassay

Male and female mosquitoes exposed to permethrin and DDT were confirmed resistant in both sexes after 24 hours of exposure. Mosquitoes exposed to lambda-cyhalothrin showed possible resistance (Table 1).

**Table 1:** Percentage mortality of *Anopheles coluzzii* in WHO susceptibility bioassays, by insecticide and sex.

Insecticide	Male (%)	Female (%)	Average (%)
Permethrin	51	35	43
DDT	76	75	75.5
Lambda-cyhalothrin	90	94	92

Note: n = 100 per sex per insecticide

### Behavioural assessment of *Anopheles* mosquitoes to insecticides

#### Flight efficiency.

The flight efficiency of resistant and unexposed mosquito populations to selected pyrethroid and organochloride insecticides is shown (Table 2). There was generally an increase in mean flight efficiency with time in resistant mosquitoes introduced to cages with designated insecticides, including lambda-cyhalothrin, DDT, and permethrin. Mean flight efficiency time in lambda-cyhalothrin-resistant mosquitoes with test paper increased from 10 to 40 minutes and declined slightly from 50 to 60 minutes. No flight activity was recorded from 10 to 60 minutes in unexposed mosquitoes introduced to lambda-cyhalothrin test paper and in exposures without the test paper. In permethrin-resistant mosquitoes exposed to the permethrin test paper, mean flight efficiency time increased from 10 to 30 minutes and declined from 40 to 60 minutes. Mean flight efficiency increased with time in DDT-resistant mosquitoes exposed to DDT test paper. In unexposed mosquitoes introduced to DDT test papers, flight time increased from 20 to 60 minutes. Flight efficiency did not follow a regular pattern in resistant mosquitoes exposed to lambda-cyhalothrin, permethrin, or DDT without the test paper, nor in unexposed mosquitoes with or without test papers. The difference in the mean flight efficiency among the twelve exposure-condition combinations was significant (F = 18.746; p < 0.0001).

**Table 2:** Flight efficiency of resistant and unexposed mosquito populations to selected pyrethroid and organochloride insecticides.

				Mean flight efficiency time (minutes)					
Mosquito	Treatment	Conc. (%)	Exposure form	10	20	30	40	50	60
Exposed	Lambda-cyhalothrin	0.05	WITP	3.00 ± 1.41	6.50 ± 3.54	15.00 ± 2.83	24.50 ± 4.95	22.50 ± 3.54	18.50 ± 2.12
			WOITP	2.00 ± 1.41	0.50 ± 0.71	1.50 ± 0.71	1.50 ± 0.71	0.50 ± 0.71	0.50 ± 0.71
Unexposed	Lambda-cyhalothrin	0.05	WITP	0.00 ± 0.00	0.00 ± 0.00	0.00 ± 0.00	0.00 ± 0.00	0.00 ± 0.00	0.00 ± 0.00
			WOITP	0.00 ± 0.00	0.00 ± 0.00	0.00 ± 0.00	0.00 ± 0.00	0.00 ± 0.00	0.00 ± 0.00
Exposed	Permethrin	0.75	WITP	0.50 ± 0.71	13.5 ± 2.12	22.00 ± 2.83	15.5 ± 2.12	20.00 ± 1.41	14.00 ± 1.41
			WOITP	2.00 ± 1.41	0.50 ± 0.71	1.50 ± 0.71	1.50 ± 0.71	3.00 ± 1.41	0.50 ± 0.71
Unexposed	Permethrin	0.75	WITP	0.00 ± 0.00	0.50 ± 0.71	0.50 ± 0.71	1.50 ± 2.12	0.50 ± 0.71	1.00 ± 1.41
			WOITP	0.00 ± 0.00	0.00 ± 0.00	0.50 ± 0.71	0.00 ± 0.00	1.00 ± 1.41	0.50 ± 0.71
Exposed	DDT	4.00	WITP	6.00 ± 1.41	11.00 ± 1.41	14.5 ± 2.12	20.50 ± 2.12	28.00 ± 1.41	30.50 ± 0.71
			WOITP	1.50 ± 0.71	1.00 ± 0.00	3.00 ± 1.41	2.50 ± 0.71	0.50 ± 0.71	1.50 ± 0.71
Unexposed	DDT	4.00	WITP	1.00 ± 1.41	0.00 ± 0.00	0.50 ± 0.71	0.50 ± 0.71	2.00 ± 1.41	3.50 ± 2.12
			WOITP	0.50 ± 0.71	0.00 ± 0.00	0.00 ± 0.00	1.00 ± 1.41	0.00 ± 0.00	0.00 ± 0.00

**Note:** Values are mean ± standard deviation. WITP means with insecticide treated paper, WOITP means without insecticide treated paper

The percentage flight efficiency of resistant and unexposed mosquitoes introduced to designated insecticide test papers is also shown (Fig 2). DDT-resistant mosquitoes exposed to DDT test paper had the highest flight efficiency (100%) in 60 minutes. Mosquitoes in other exposures were below 40% at 60 minutes. The difference in percentage flight efficiency was significant (p = 0.017).

**Fig 2:**
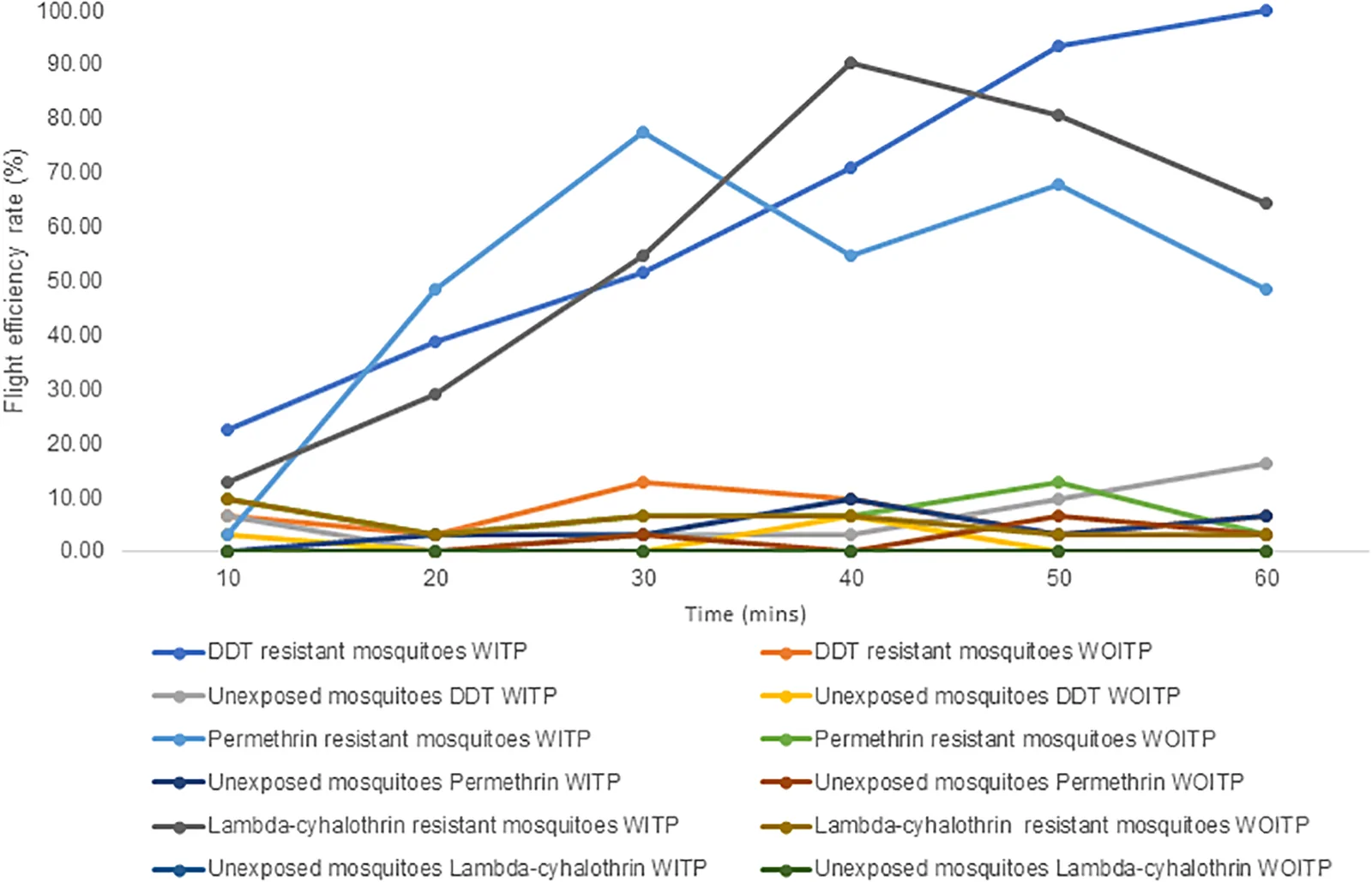
Percentage flight efficiency of exposed and unexposed mosquitoes introduced to designated insecticide test papers. [percentage-transformed values; twelve exposure-condition combinations] (F = 3.063; p = 0.017). **Legend:** WITP means with insecticide test paper, WOITP means without insecticide test paper.

#### Resting efficiency.

The resting efficiency of resistant and unexposed mosquito populations to selected pyrethroid and organochloride insecticides is shown (Table 3). Resting efficiency time was higher in resistant mosquito populations without lambda-cyhalothrin, permethrin, and DDT test papers. Likewise, the same trend was observed in unexposed mosquitoes not introduced to lambda-cyhalothrin, permethrin, and DDT test papers. The resting efficiency rate in these exposures did not follow a regular pattern of increase with time. Resting of mosquitoes was steady in unexposed mosquitoes without lambda-cyhalothrin test paper (mean of 15.00). In resistant mosquitoes exposed to lambda-cyhalothrin test paper, resting time decreased from 10 minutes to 20 minutes and from 30 minutes to 60 minutes; there was no resting during exposure. No resting was observed from 10 to 60 minutes in unexposed mosquitoes introduced to lambda-cyhalothrin test papers. In permethrin-resistant mosquitoes exposed to permethrin test paper, there was a decline in resting efficiency (mean of 12.5) at 10 minutes and no rest from 20 to 60 minutes. In DDT-resistant mosquitoes, resting efficiency decreased from a mean of 6.50 to a mean of 2.00 in 20 minutes, and from 30 to 60 minutes no resting occurred. The difference in mean resting efficiency among the twelve exposure-condition combinations was significant (F = 36.265; p < 0.0001).

**Table 3:** Resting efficiency of resistant and unexposed mosquito populations to selected pyrethroid and organochloride insecticides.

				Mean resting efficiency time (minutes)					
Mosquito	Treatment	Conc. (%)	Exposure form	10	20	30	40	50	60
Exposed	Lambda-cyhalothrin	0.05	WITP	10.00 ± 1.41	5.00 ± 1.41	0.00 ± 0.00	0.00 ± 0.00	0.00 ± 0.00	0.00 ± 0.00
			WOITP	11.50 ± 0.71	13.00 ± 1.41	12.00 ± 1.41	12.00 ± 1.41	13.50 ± 0.71	13.50 ± 0.71
Unexposed	Lambda-cyhalothrin	0.05	WITP	0.00 ± 0.00	0.00 ± 0.00	0.00 ± 0.00	0.00 ± 0.00	0.00 ± 0.00	0.00 ± 0.00
			WOITP	15.00 ± 0.00	15.00 ± 0.00	15.00 ± 0.00	15.00 ± 0.00	15.00 ± 0.00	15.00 ± 0.00
Exposed	Permethrin	0.75	WITP	12.5 ± 2.12	0.00 ± 0.00	0.00 ± 0.00	0.00 ± 0.00	0.00 ± 0.00	0.00 ± 0.00
			WOITP	11.50 ± 0.71	13.00 ± 1.41	12.00 ± 1.41	12.00 ± 1.41	9.50 ± 2.12	13.00 ± 1.41
Unexposed	Permethrin	0.75	WITP	1.50 ± 2.12	1.50 ± 2.12	1.50 ± 2.12	1.50 ± 2.12	1.50 ± 2.12	1.50 ± 2.12
			WOITP	6.00 ± 8.49	6.00 ± 8.49	6.00 ± 8.49	6.00 ± 8.49	6.00 ± 8.49	6.00 ± 8.49
Exposed	DDT	4.00	WITP	6.50 ± 2.12	2.00 ± 1.41	0.00 ± 0.00	0.00 ± 0.00	0.00 ± 0.00	0.00 ± 0.00
			WOITP	12.00 ± 1.41	13.00 ± 1.41	10.00 ± 1.41	11.00 ± 1.41	12.50 ± 2.12	12.50 ± 0.71
Unexposed	DDT	4.00	WITP	0.00 ± 0.00	0.00 ± 0.00	0.50 ± 0.71	0.50 ± 0.71	0.50 ± 0.71	0.50 ± 0.71
			WOITP	14.50 ± 0.71	14.50 ± 0.71	13.50 ± 0.71	13.50 ± 0.71	13.50 ± 0.71	13.50 ± 0.71

**Note:** Values are mean ± standard deviation. WITP means with insecticide treated paper, WOITP means without insecticide treated paper

The percentage resting efficiency of resistant and unexposed mosquitoes introduced to designated insecticide test papers is shown in Fig 3. There was a high percentage resting rate in the unexposed mosquito group with or without test papers and in the resistant mosquito group without test papers. The difference in resting efficiency over time was significant (p < 0.05).

**Fig 3:**
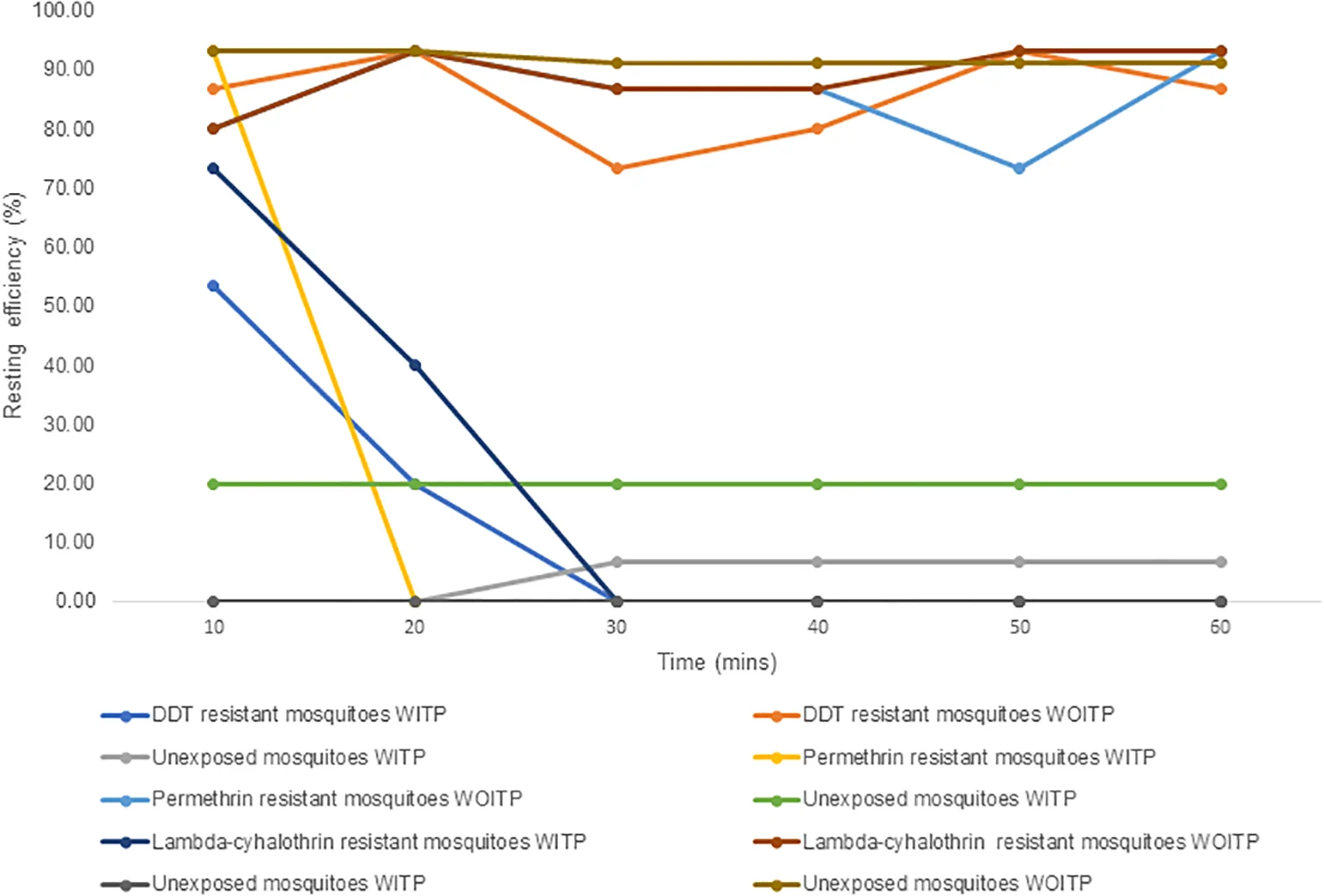
Percentage resting efficiency of exposed and unexposed mosquitoes introduced to designated insecticide test papers. [percentage-transformed values; twelve exposure-condition combinations — all 12 combinations now plotted] (F = 2.652; p = 0.035). **Legend:** WITP means with insecticide test paper, WOITP means without insecticide test paper.

#### Repellency of mosquitoes.

The repellency of *Anopheles* mosquitoes to selected pyrethroid and organochloride insecticides using cross-framed chambers is shown (Table 4). There was higher crossover of mosquitoes from the zone of resistant mosquitoes exposed to the designated insecticide test papers than in the unexposed mosquito group introduced to the test papers. There was a sharper decline in the mosquito population in the resistant mosquito group exposed to the designated insecticide test papers than in the unexposed group. The unexposed mosquito population introduced to lambda-cyhalothrin test paper or without introduction had steady repellency from 10 to 60 minutes. The difference in repellency among the twelve exposure-condition combinations was significant (F = 34.940; p < 0.0001). The percentage repellency of resistant and unexposed mosquitoes introduced to designated insecticide test papers is shown in Fig 4. There was a sharp decline in the percentage of mosquitoes repelled in the resistant mosquito group. The difference was significant with time (p < 0.05).

**Table 4:** Repellency of *Anopheles* mosquitoes to selected pyrethroid and organochloride insecticide using cross-framed chambers.

				Mean repellency (escape count per 10-minute interval)					
Mosquito	Treatment	Conc. (%)	Exposure form	10	20	30	40	50	60
Exposed	Lambda-cyhalothrin	0.05	WITP	14.00 ± 1.41	13.00 ± 1.41	9.50 ± 0.71	7.50 ± 0.71	5.00 ± 1.41	4.50 ± 0.71
			WOITP	0.00 ± 0.00	0.50 ± 0.71	3.00 ± 1.41	1.50 ± 0.71	1.50 ± 0.71	0.50 ± 0.71
Unexposed	Lambda-cyhalothrin	0.05	WITP	11.50 ± 0.71	11.50 ± 0.71	11.50 ± 0.71	11.50 ± 0.71	11.50 ± 0.71	11.50 ± 0.71
			WOITP	2.00 ± 1.41	2.00 ± 1.41	2.00 ± 1.41	2.00 ± 1.41	2.00 ± 1.41	2.00 ± 1.41
Exposed	Permethrin	0.75	WITP	10.00 ± 1.41	8.00 ± 1.41	6.00 ± 1.41	5.50 ± 0.71	2.00 ± 1.41	0.00 ± 0.00
			WOITP	3.00 ± 1.41	1.00 ± 1.41	1.50 ± 0.71	0.50 ± 0.71	2.00 ± 1.41	2.00 ± 2.12
Unexposed	Permethrin	0.75	WITP	14.50 ± 0.71	13.50 ± 0.71	13.50 ± 0.71	13.50 ± 0.71	13.50 ± 0.71	13.50 ± 0.71
			WOITP	0.00 ± 0.00	0.50 ± 0.71	0.50 ± 0.71	0.50 ± 0.71	0.50 ± 0.71	0.50 ± 0.71
Exposed	DDT	4.00	WITP	11.00 ± 1.41	9.00 ± 1.41	4.00 ± 1.41	3.50 ± 0.71	1.50 ± 0.71	0.50 ± 0.71
			WOITP	2.50 ± 0.71	1.50 ± 0.71	3.50 ± 0.71	1.00 ± 0.00	1.00 ± 1.41	0.50 ± 0.71
Unexposed	DDT	4.00	WITP	14.50 ± 0.71	14.50 ± 0.71	14.50 ± 0.71	14.50 ± 0.71	14.50 ± 0.71	14.50 ± 0.71
			WOITP	0.00 ± 0.00	0.00 ± 0.00	0.00 ± 0.00	0.50 ± 0.71	0.50 ± 0.71	1.50 ± 0.71

**Note:** Values are mean ± standard deviation. WITP means with insecticide treated paper, WOITP means without insecticide treated paper

**Fig 4:**
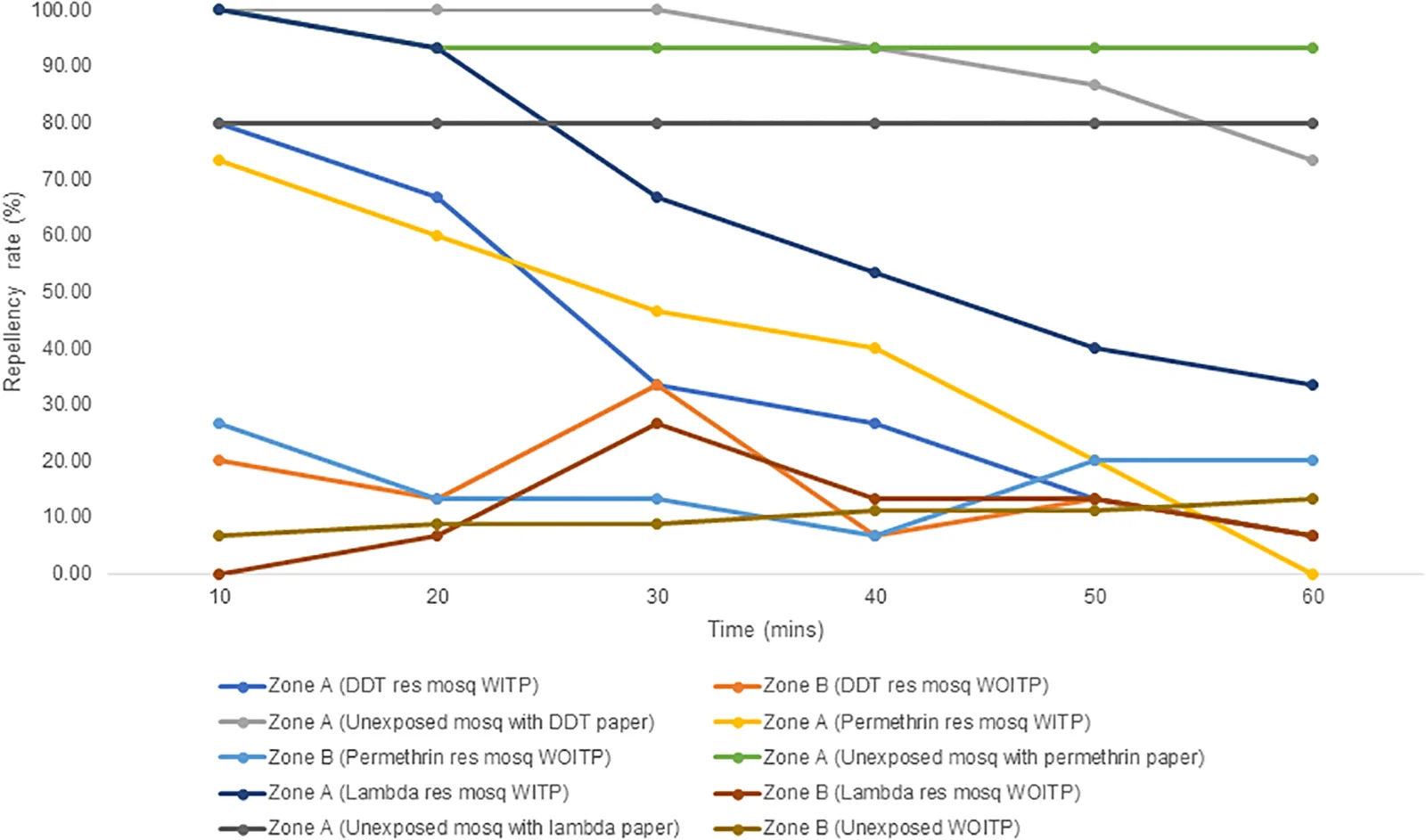
Percentage repellency of resistant and unexposed mosquitoes introduced to designated insecticide test papers. [all twelve exposure-condition combinations shown] (F = 4.600; p = 0.002). **Legend:** WITP means with insecticide test paper, WOITP means without insecticide test paper, Lambda means lambda-cyhalothrin, res means resistant and mosq means mosquito.

#### Landing and biting efficiency.

The landing and biting efficiency of *Anopheles* mosquitoes to selected pyrethroid and organochloride insecticides is presented (Table 5). The mean biting rate was reduced in females exposed to the different insecticides compared to the control. A similar trend was observed for mosquito landing on the insecticide-treated surface. Landing and biting were highest in female mosquitoes in the control group at 20 minutes. Landing and biting decreased with time after 20 minutes in the control group. Landing and biting in female mosquitoes exposed to lambda-cyhalothrin were higher at 20 minutes compared to others. Mean landing and biting were higher for females exposed to permethrin at 10 minutes (mean of 1.50 respectively). In DDT-exposed female mosquitoes, mean landing was highest at 30 minutes, and biting was highest at 40 minutes. Generally, initiation of landing started at 10 minutes in female mosquitoes exposed to the insecticide-treated surface, and the highest at 10 minutes occurred with females exposed to lambda-cyhalothrin. Likewise, mean biting was initiated at 10 minutes in all exposures except in female mosquitoes exposed to lambda-cyhalothrin and DDT. In DDT and lambda-cyhalothrin, biting was initiated at 20 minutes. The difference in biting rate among the four treatment groups (control, lambda-cyhalothrin, permethrin, DDT) was significant (F = 6.455, p < 0.0001). Even though there were landings by male mosquitoes, no biting occurred in any exposure. The difference in landing among the four treatment groups (control, lambda-cyhalothrin, permethrin, DDT) was significant (F = 11.572, p < 0.0001). Although male mosquitoes landed on the insecticide-treated surface in all exposure groups, no biting was recorded in any male across all treatments, confirming that only female Anopheles mosquitoes engage in blood-feeding behaviour, consistent with their established role in disease transmission.

**Table 5:** Landing and biting efficiency of *Anopheles* mosquitoes to selected pyrethroid and organochloride insecticides.

				Mean landing/biting count (per 10-minute interval)					
Behaviour	Treatment	Conc. (%)	Sex	10	20	30	40	50	60
Biting	Control	0.00	Female	1.50 ± 0.71	4.00 ± 1.41	1.50 ± 0.71	1.50 ± 0.71	0.00 ± 0.00	1.50 ± 0.71
			Male	0.00 ± 0.00	0.00 ± 0.00	0.00 ± 0.00	0.00 ± 0.00	0.00 ± 0.00	0.00 ± 0.00
Landing	Control	0.00	Female	1.50 ± 0.71	6.00 ± 1.41	2.50 ± 0.71	1.50 ± 0.71	3.00 ± 1.41	2.00 ± 1.41
			Male	0.50 ± 0.71	0.00 ± 0.00	0.00 ± 0.00	0.00 ± 0.00	0.50 ± 0.71	0.50 ± 0.71
Biting	Lambda-cyhalothrin	0.05	Female	0.00 ± 0.00	0.50 ± 0.71	0.00 ± 0.00	0.00 ± 0.00	0.00 ± 0.00	0.50 ± 0.71
			Male	0.00 ± 0.00	0.00 ± 0.00	0.00 ± 0.00	0.00 ± 0.00	0.00 ± 0.00	0.00 ± 0.00
Landing	Lambda-cyhalothrin	0.05	Female	2.00 ± 1.41	2.50 ± 2.12	0.50 ± 0.71	1.50 ± 0.71	1.00 ± 1.41	1.00 ± 1.41
			Male	1.50 ± 0.71	0.50 ± 0.71	0.00 ± 0.00	0.00 ± 0.00	3.00 ± 1.41	0.00 ± 0.00
Biting	Permethrin	0.75	Female	1.50 ± 0.71	0.50 ± 0.71	0.50 ± 0.71	0.50 ± 0.71	0.50 ± 0.71	0.50 ± 0.71
			Male	0.00 ± 0.00	0.00 ± 0.00	0.00 ± 0.00	0.00 ± 0.00	0.00 ± 0.00	0.00 ± 0.00
Landing	Permethrin	0.75	Female	1.50 ± 0.71	0.50 ± 0.71	0.50 ± 0.71	0.50 ± 0.71	0.50 ± 0.71	0.50 ± 0.71
			Male	0.50 ± 0.71	0.50 ± 0.71	0.00 ± 0.00	0.00 ± 0.00	0.00 ± 0.00	0.00 ± 0.00
Biting	DDT	4.00	Female	0.00 ± 0.00	1.50 ± 0.71	0.00 ± 0.00	2.00 ± 1.41	0.00 ± 0.00	0.00 ± 0.00
			Male	0.00 ± 0.00	0.00 ± 0.00	0.00 ± 0.00	0.00 ± 0.00	0.00 ± 0.00	0.00 ± 0.00
Landing	DDT	4.00	Female	1.00 ± 1.41	0.50 ± 0.71	2.00 ± 1.41	1.00 ± 1.41	1.00 ± 1.41	0.50 ± 0.71
			Male	0.50 ± 0.71	0.00 ± 0.00	0.00 ± 0.00	0.00 ± 0.00	0.00 ± 0.00	0.00 ± 0.00

**Note:** Values are mean ± standard deviation.

The percentage landing and biting of mosquitoes exposed to selected pyrethroid and organochloride insecticides is shown (Fig 5 and 6, respectively). Landing was higher in the control group and peaked at 20 minutes. DDT-resistant females followed closely, with a peak at 30 minutes. The difference over time was not significant (p > 0.05). In control females, percentage bites peaked at 20 minutes and decreased up to 50 minutes, increasing again at 60 minutes. There were double peaks in biting for DDT-resistant females at 20 minutes and 40 minutes, respectively. The difference with time was not significant (p > 0.05).

**Fig 5:**
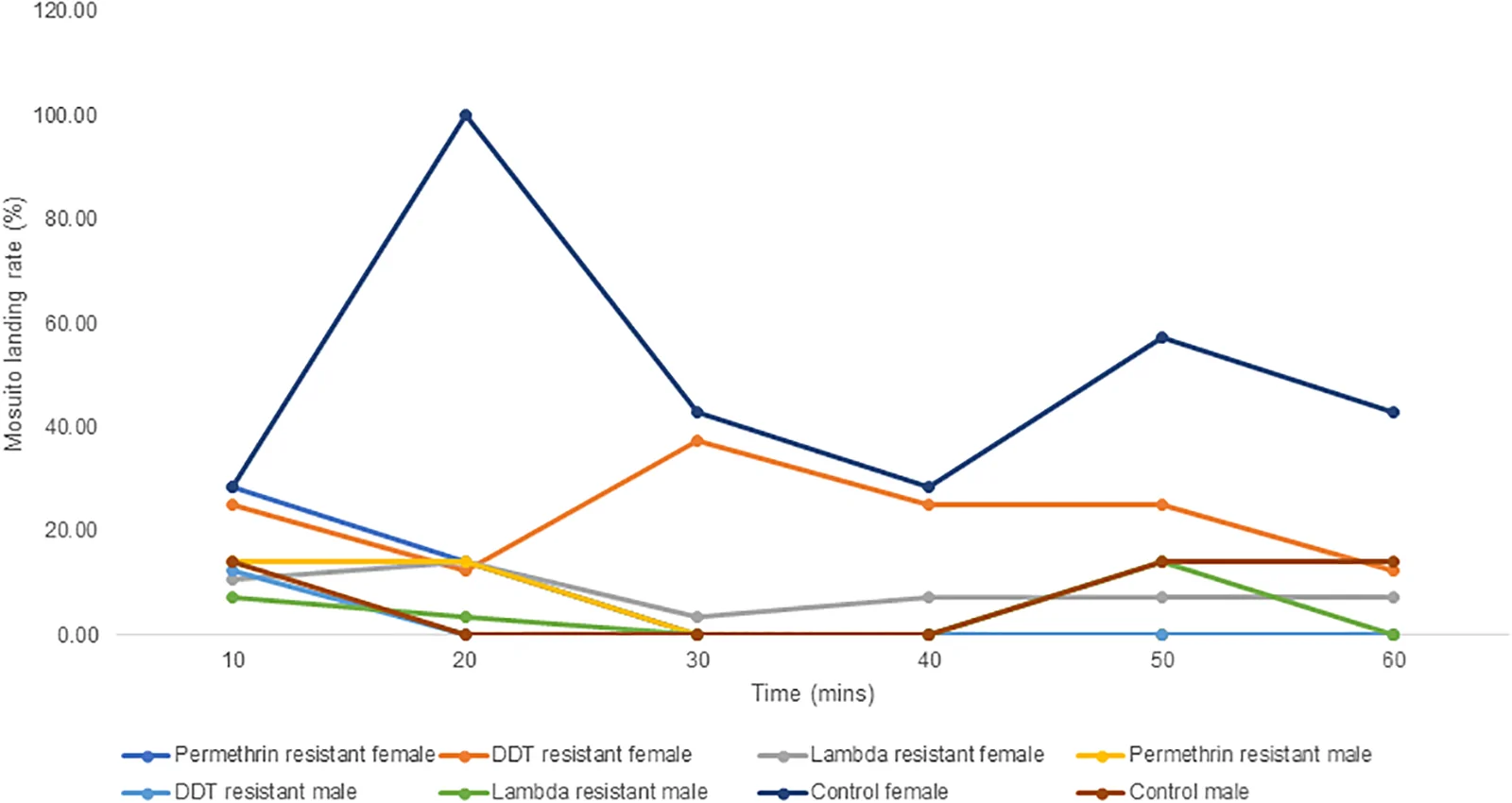
Percentage landing of mosquitoes exposed to selected pyrethroid and organochloride insecticides. [percentage-transformed values; four treatment groups] (F = 1.401; p = 0. 248).

**Fig 6:**
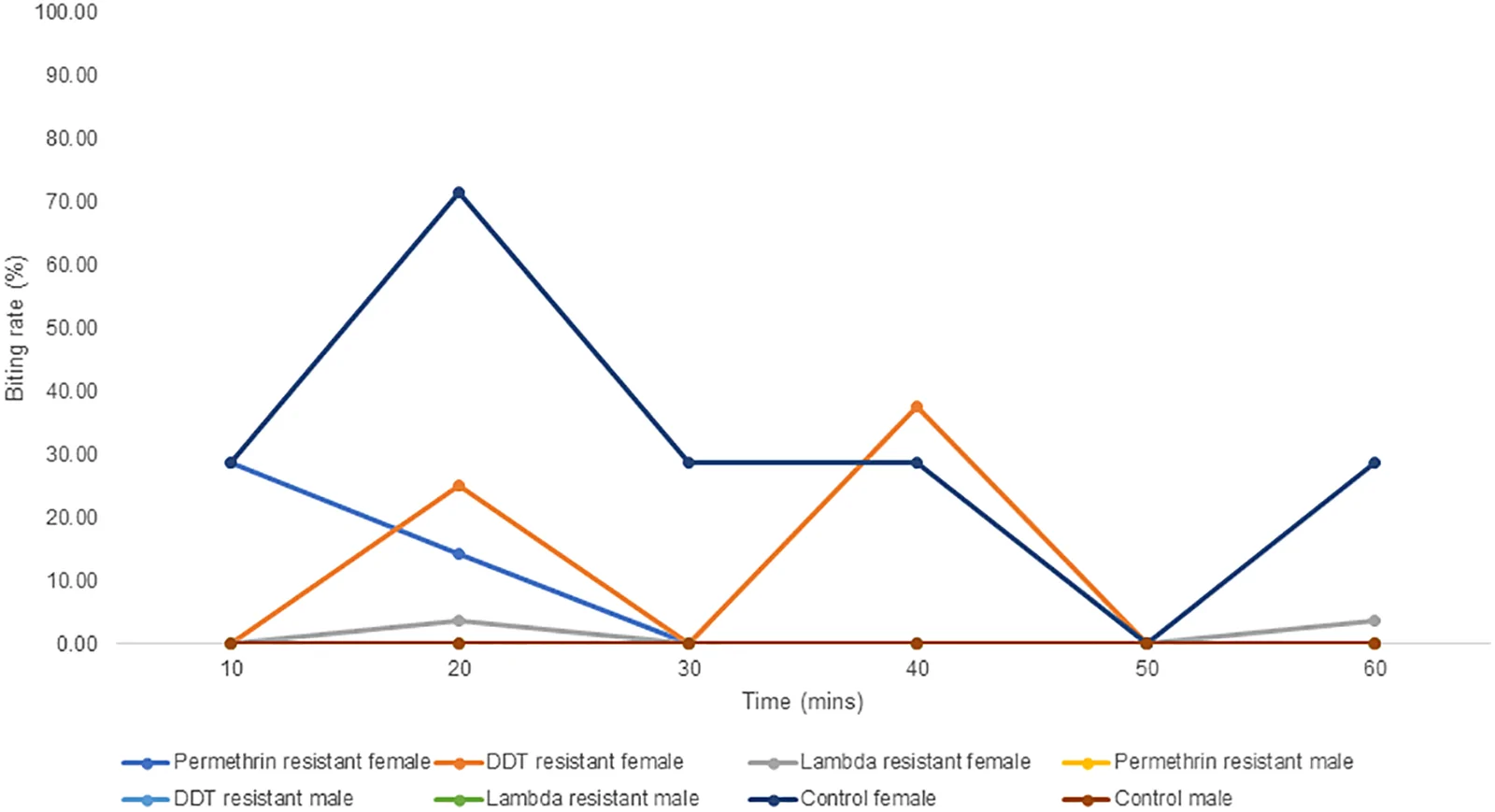
Percentage biting of mosquitoes exposed to selected pyrethroid and organochloride insecticides. [percentage-transformed values; four treatment groups] (F = 1.794; p = 0.140).

#### Oviposition fitness.

The feeding proportion of resistant female mosquitoes exposed to selected pyrethroid and organochloride and control is shown (Fig 7). The proportion of blood-fed mosquitoes was highest in unexposed females. This was closely followed by DDT resistant blood fed mosquitoes. The least blood-fed mosquitoes occurred in lambda-cyhalothrin. Half fed mosquito was highest in DDT resistant mosquitoes. The other female-resistant mosquitoes had equal numbers of half-fed mosquitoes. Unfed mosquito was highest for lambda-cyhalothrin-resistant females and closely followed by permethrin resistant females. Unexposed female mosquitoes recorded no unfed mosquitoes.

**Fig 7:**
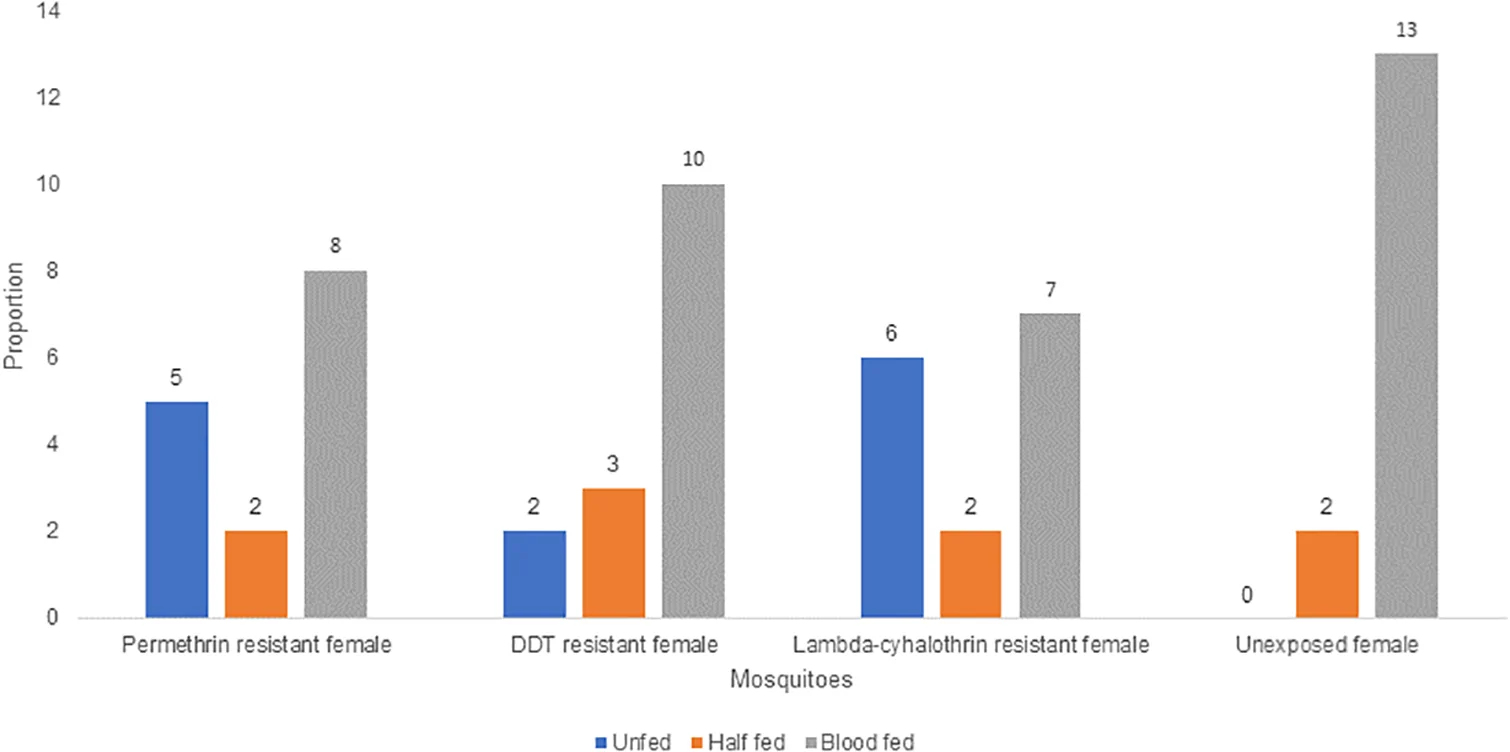
Feeding proportion of resistant female mosquitoes exposed to selected pyrethroid and organochloride and control.

The oviposition success of crossed mosquitoes exposed to the selected pyrethroid and organochloride insecticides with unexposed mosquitoes is shown (Table 6). Five female and five male mosquitoes were used in each crossing. The highest oviposition was recorded in unexposed males and females (mean of 0.33). There was no oviposition in the other crossings (mean of 0.00).

**Table 6:** Oviposition fitness of crossed mosquitoes exposed to the selected pyrethroid and organochloride insecticides compared with unexposed mosquitoes.

Reproductive cross	No. mosquitoes	No. egg	Mean eggs ± SD
UN EX M + UN EX Female	5	12	0.33 ± 0.58
PERMETHRIN EX F + UN EX M	5	0	0.00 ± 0.00
PERMETHRIN EX M + UN EX F	5	0	0.00 ± 0.00
LAMBDA EX F + UN EX M	5	0	0.00 ± 0.00
LAMBDA EX M + UN EX F	5	0	0.00 ± 0.00
DDT EX F + UN EX M	5	0	0.00 ± 0.00
DDT EX M + UN EX F	5	0	0.00 ± 0.00

**Note:** Values are means of eggs ± standard deviation; EX F means exposed female, UN EX F means unexposed female, EX M means exposed male, UN EX M means unexposed male.

## Discussion

This study demonstrates that mosquitoes resistant to selected pyrethroids maintained measurable flight activity compared to the unexposed mosquitoes, which exhibited negligible flight. Mosquitoes resistant to lambda-cyhalothrin showed a progressive increase in mean flight duration with time of exposure up to 40 minutes of exposure periodicity, with a slight decline following. Contrasting that, in unexposed mosquitoes, mosquitoes remained largely inactive throughout the observation period. This pattern suggests that even though resistant mosquitoes survive insecticide exposure, they experience altered locomotor activity, reflecting sublethal behavioural effects rather than complete incapacitation. It should be noted that the unexposed (control) group was not individually screened for resistance status before testing, and therefore likely comprised a mixture of susceptible and resistant individuals; consequently, some of the reduced activity observed in this group may be attributable to natural variation in baseline activity or underlying physiological differences rather than solely reflecting an absence of insecticide-induced effects. There are similar observations reported in resistant *Anopheles* populations, where exposure to pyrethroids or DDT induces hyperactivity followed by reduced flight efficiency [18,19]. The initial increase in activity is consistent with irritancy-induced excitation, while the subsequent decline reflects neuromuscular fatigue [5]. These findings suggest that sublethal exposure may alter dispersal patterns and host-seeking behaviour in resistant mosquitoes, potentially affecting malaria transmission dynamics.

Resting behaviour is common in blood fed mosquitoes or after prolonged flight with distance. The assessment of resting efficiency in pyrethroid resistant mosquitoes show that there was significantly reduced resting with time on the insecticide-treated surfaces. Resistant mosquitoes exhibited reduced resting as time progressed to complete avoidance of surfaces from 30 minutes onward compared to the unexposed mosquitoes that maintained stable resting times. This behaviour aligns with contact irritancy and excito-repellency observed in pyrethroid-resistant *Anopheles* mosquitoes [10,11]. Avoidance of treated surfaces reduces insecticide uptake, thereby enhancing survival. The observed behavioural resistance complements physiological mechanisms such as metabolic detoxification and kdr mutations, amplifying resistance effects [6,19].

Mosquitoes exhibit an exit tendency toward unfavorable environments and/or after successful digestion of a blood meal. Repellency assessment showed that insecticide-treated papers deterred mosquitoes, as reflected in increased exit behavior. Resistant mosquitoes exposed to permethrin-treated papers exhibited a progressive decrease in knockdown time due to rapid movement away from treated surfaces, whereas unexposed mosquitoes maintained stable responses. These findings corroborate studies showing that pyrethroids and DDT exert both toxic and behavioral effects, including irritancy-induced escape [5]. Behavioral avoidance limits contact with treated surfaces, potentially reducing insecticide exposure but also promoting shifts in feeding or resting locations that sustain malaria transmission [7,10].

Landing and biting efficiency were reduced in mosquitoes exposed to insecticides. Female mosquitoes resistant to lambda-cyhalothrin showed lower landing rates compared to the unexposed group, and biting activity was similarly reduced. Male mosquitoes displayed landing behaviour with no biting, consistent with their non-hematophagous nature. This reduction suggests that insecticide exposure disrupts host-seeking behaviour, likely through neurotoxic effects impairing sensory perception and motor coordination [11,18]. Although lower biting frequency could reduce immediate transmission, resistant mosquitoes may compensate through behavioural resistance mechanisms such as increased feeding attempts or altered feeding times [5].

Oviposition fitness demonstrated a marked reduction in reproductive output following insecticide exposure. The unexposed females produced measurable egg clusters, whereas all exposed groups recorded no oviposition, reflecting substantial impairment. Blood-feeding success was also reduced. These findings indicate a strong fitness cost associated with insecticide exposure, consistent with studies linking metabolic and physiological stress to reduced reproductive performance in resistant populations [17,18]. The complete absence of oviposition in this study is more pronounced than partial reductions reported elsewhere, possibly reflecting strain-specific responses or exposure intensity [10].

The cross-mating experiments revealed that insecticide exposure significantly impaired reproductive success. Crosses involving exposed females, regardless of male status, produced no eggs, whereas unexposed crosses resulted in normal oviposition. This demonstrates that insecticide exposure affects both female physiology and male-mediated reproductive processes such as insemination and sperm viability [19,31]. Altered male behaviours, including changes in flight and resting patterns, may further reduce mating opportunities, affecting population dynamics and the spread of resistance traits. These results highlight the importance of incorporating both sexes in studies of behavioural and reproductive effects of insecticide resistance [5,7,32–35].

## Conclusion

This study demonstrates that resistant and unexposed *Anopheles* mosquitoes differ in flight, resting, repellency, landing, and biting behaviours when exposed to insecticide-treated papers, suggesting that behavioural modification may be associated with insecticide resistance in this population; however, as this study compared resistant and unexposed cohorts at a single time point rather than evaluating behaviour longitudinally, these findings should not be interpreted as evidence that behavioural adaptation drives the evolution of resistance. Flight efficiency increased as resting behaviour decreased when further exposed to insecticide-treated papers. Repellency, landing, and biting activities of mosquitoes were haphazard and did not follow a regular pattern. Blood feeding decreased in exposed mosquitoes. Interestingly, this study revealed that insecticide exposure of mosquitoes affected oviposition fitness when crossed with unexposed. The induction of behavioural avoidance and reproductive impairment in resistant *Anopheles coluzzii* when exposed to insecticides emphasizes the importance of incorporating behavioural endpoints into resistance monitoring programs. These findings are key in policymaking, as they will drive vector control actions to enacting studies on reproductive gene drive.

## Supporting information

S1 Data PLoS 26 Supplementary data. (XLSX)
